# Analysis of cell cycle-related proteins in gastric intramucosal differentiated-type cancers based on mucin phenotypes: a novel hypothesis of early gastric carcinogenesis based on mucin phenotype

**DOI:** 10.1186/1471-230X-10-55

**Published:** 2010-06-07

**Authors:** Tamotsu Sugai, Mitsunori Tsukahara, Masaki Endoh, Yoshihiro Shioi, Noriko Takebe, Yoshiharu Mue, Hiroo Matsushita, Minoru Toyota, Kazuyuki Suzuki

**Affiliations:** 1 Division of Diagnostic Molecular Pathology, Department of Pathology, School of Medicine, Iwate Medical University, 19-1, Morioka City, 020-8505, Japan; 2 Division of Gastroenterology and Hepatology, Department of Internal Medicine. School of Medicine, Iwate Medical University, 19-1, Morioka City, 020-8505, Japan; 3 Division of Diabetes and Metabolism, Department of Internal Medicine, School of Medicine, Iwate Medical University, 19-1, Morioka City, 020-8505, Japan; 4 Department of Surgery, Health Insurance Hitoyoshi General Hospital, 35 Oigamicho, Hotoyoshi City, Japan; 5 Department of Biochemistry, Sapporo Medical University, 17 Sapporo City, 160-8556, Japan

## Abstract

**Background:**

Abnormalities of cell cycle regulators are common features in human cancers, and several of these factors are associated with the early development of gastric cancers. However, recent studies have shown that gastric cancer tumorigenesis was characterized by mucin expression. Thus, expression patterns of cell cycle-related proteins were investigated in the early phase of differentiated-type gastric cancers to ascertain any mechanistic relationships with mucin phenotypes.

**Methods:**

Immunostaining for Cyclins D1, A, E, and p21, p27, p53 and β-catenin was used to examine impairments of the cell cycle in 190 gastric intramucosal differentiated-type cancers. Mucin phenotypes were determined by the expressions of MUC5AC, MUC6, MUC2 and CD10. A Ki-67 positive rate (PR) was also examined.

**Results:**

Overexpressions of p53, cyclin D1 and cyclin A were significantly more frequent in a gastric phenotype than an intestinal phenotype. Cyclin A was overexpressed in a mixed phenotype compared with an intestinal phenotype, while p27 overexpression was more frequent in an intestinal phenotype than in a mixed phenotype. Reduction of p21 was a common feature of the gastric intramucosal differentiated-type cancers examined.

**Conclusions:**

Our results suggest that the levels of some cell cycle regulators appear to be associated with mucin phenotypes of early gastric differentiated-type cancers.

## Background

Progression through the cell cycle and cellular proliferation are under the control of a series of cyclins and cyclin-dependent kinase (cdk) complexes [1-3]. Accumulating evidence shows that the progression of tumorigenesis frequently involves abnormalities in the expressions of cyclins and other cell-cycle related genes [1-3]. Abnormalities have been found for cyclins D1, A, E and their co-operating partners, such as cyclin-dependent kinase (cdk), that promote cell cycle progression [1,3]. Additionally, these progressive factors can be inhibited by blockers, such as p21, p27 and p57, and another group of inhibitor proteins, including p16, p15 and p18 [4-10]. The uncontrolled proliferation that characterizes tumor cells can be largely explained by the gain and/or loss of protein functions that comprise the cell cycle. Regulation of these cell cycle-related proteins is also governed by other factors, including p53 and β-catenin, and their alterations also impair the cell cycle, resulting in uncontrolled proliferation [11-15].

Of the above cell cycle-related proteins, key regulators of progression through the G1 phase of the cell cycle are cyclin D1 and cyclin E, p53, p21 and p27 [1,4,6,12]. Their abnormal expressions have been thought to play pivotal roles in the progression of tumorigenesis and have been found to be disturbed in a number of human malignancies. Cyclin A is also a member of the cyclin protein superfamily that can be activated during the transition from the G1 to the S phase of the cell cycle. Abnormal expressions of cyclin A are correlated with poor outcomes in various human cancers [8,9]. In addition, nuclear expression of β-catenin is implicated in gastrointestinal cancers [14,15]. β-catenin accumulates in the nucleus due to impairments of the Wnt signal pathway, and its nuclear expression promotes progression of the cell cycle and cellular proliferation [14,15]. However, to date, its activity has not been shown to affect the pathogenesis of early differentiated-type gastric cancers.

Recent studies have shown that cellular mucin expressions and tumor phenotypes are associated with the clinico-pathological findings and tumorigenesis in differentiated-type gastric cancers [16-19]. The mucin phenotypes of tumors have been primarily classified into 3 types: gastric, intestinal and mixed phenotypes [16,17]. The gastric phenotype is characterized by poor outcomes, distinct histological features and a specific subtype of genetic alterations, including microsatellite instability (MSI) [17,18]. In contrast, the intestinal phenotype is a very well differentiated type, with low proliferative activity and a lack of MSI [17]. The expressions of mucins by tumor cells define tumor characteristics in gastric cancers [16-19]. Thus, it is important for the understanding of early tumorigeneis of gastric cancers to examine biological alterations according to these mucin phenotypes [16-19].

Although a number of studies regarding the expressions of cell cycle-related factors have been reported [3-7], the associations of early differentiated-type gastric cancers and their mucin phenotypes and alterations of cell-cycle-related proteins are not fully understood. In the present study, we examined abnormalities of cell cycle-related proteins of the early phase of differentiated-type gastric cancers based on mucin phenotypes.

## Methods

### Patients

Materials for this study were obtained from 190 patients with primary early gastric cancers that were diagnosed at the Division of Molecular Diagnostic Pathology, Department of Pathology, Iwate Medical University, Morioka, Japan. Informed consent was given in all patients that we examined. In addition, our study was approved by our ethics committee (title, molecular analysis of gastrointestinal tumors and the surrounding mucosa; reference number, H21-140, ethics committee of Iwate Medical University). These tumors were consistent with intramucosal differentiated-type cancers, and were obtained from samples of endoscopic submucosal dissection (ESD). The histological criteria used to make histological diagnoses were based on our hospital criteria [16], which were modified from those of the Japanese Research Society for Gastric Cancers [20], as discrepancies in histological judgments exist between Japanese and Western pathologists. Detailed clinico-pathological data (patient age, sex, site of tumor, tumor size, macroscopic type and degree of tumor differentiation) are shown in Additional file 1.

In this study, intramucosal differentiated-type cancers were sub-classified into 3 groups according to the tumor nuclear grade: low, intermediate or high. The tumor grade was determined according to previously published criteria [17]. In brief, low-grade cancer was diagnosed by the presence of cells with enlarged, hyperchromatic nuclei that were largely confined to the basal portions of the cells, but that had more irregular and complex glands than gastric adenomas. Tumors that contained cells having an obvious loss of polarity, high nuclear irregularity, enlarged nucleolus and hyperchromatin were classified as high-grade cancers. The high grade cancers often showed significant architectural atypia, such as high architectural or glandular distortions. Intermediate findings between low and high-grade cancers were recognized as intermediate cancers. However, the histological distinction between gastric adenoma and low-grade cancer was sometimes very difficult. The slides were independently evaluated by 2 experienced pathologists (S. T. and N. S.); in some cases in which the evaluations provided different results, a consensus interpretation was reached after re-examination.

### Immunohistochemistry

Immediately after excision, specimens were fixed in 20% neutral buffered formalin, embedded in paraffin wax, cut into 3 μm paraffin sections and stained with haematoxylin and eosin (HE) for routine light microscopy. For immunohistochemical staining, additional 3 μm thick sections were cut from paraffin-embedded tissue and placed on poly-L-lysine-coated glass slides. In brief, sections were deparaffinized in xylene and dehydrated. For the determination of mucin phenotypes, immunostaining was done for MUC 2 (Ccp58, Novocastra Laboratories, Newcastle, UK), CD10 (56C6, Novocastra Laboratories), MUC 5AC (CLH2, Novocastra Laboratories) and MUC 6 (CLH5, Novocastra Laboratories, Newcastle, UK). In addition, immunostaining was done for p53 (clone, DO7, DAKO, Carpinteria, CA, USA), Ki-67 (MIB-1, Dako), p21 (SX118, Dako), p27 (SX53G8, Dako), cyclin D1 (SP4, Nichirei Co., Tokyo), cyclin E (13A3, Novocastra Laboratories), cyclin A (6E6, Novocastra Laboratories) and β-catenin (BD, Transduction Laboratories).

Immunohistochemistry used the DAKO Envision+ system, consisting of dextran polymers conjugated with horseradish peroxidase (DAKO), as previously described [16,17]. The specimens were heated by microwave in citrate buffer (pH 6.0) 3 times for 5 min each at 750W before the reactions with antibodies (H2500, Microwave Processor, Bio Rad, USA), as previously described [16,17]. Hematoxylin was used as the counterstain.

### Evaluation of mucin expression

In this study, gastric tumors were classified into 4 groups according to their immunostaining patterns. A gastric phenotype was defined as tumors having predominant intracytoplasmic mucin of the gastric type, as determined by immunostaining with human gastric mucin (MUC5AC) and/or pyloric gland mucin (MUC6), but with no MUC-2 positive cells. An intestinal phenotype was indicated by a tumor showing MUC2-positive cells and/or CD10 positive (along with a brush border). These tumors were differentiated to small intestinal absorptive cells. Intestinal phenotype tumors were sub-classified into 2 groups: colonic type (positive for MUC 2 only) and small intestinal type (positive for CD10). A mixed phenotype was defined as a tumor having immunostaining consistent with gastric type (positive cells for MUC5AC and/or pyloric gland mucin) as well as intestinal type mucin (positive cells for CD10 and/or MUC2). Finally, tumors without immunostaining for either gastric or intestinal phenotypes were assigned to an "unclassified" type. Immunopositive results for > 5% of the tumor cells were regarded as positive, and immunopositive results for < 5% as negative, in accordance with previous reports [16,17,21].

### Measuring the Ki-67 positive rate (PR)

Random areas from each sample were selected in which to count Ki-67 positive nuclei. The Ki-67 positive rate was calculated as the percentage of Ki-67 positive tumor cell nuclei determined in 10 high-power fields (HPF, × 400), which was determined after counting at least 500 tumor cells per tumor sample. Tumor proliferative activity was classified into 2 groups: high and low. The proliferative activity was determined according to our criteria [16,17]. In brief, PR of the tissue was measured in both the tumor tissue and its surrounding non-neoplastic tissue. High proliferation was considered if PR was higher in the tumor tissue than in the non-neoplastic tissue. Low proliferation was judged if PR was lower in the tumor tissue than in the non-neoplastic tissue.

### Immunohistochemical assessment of cell cycle-related proteins

**Figure 1:**
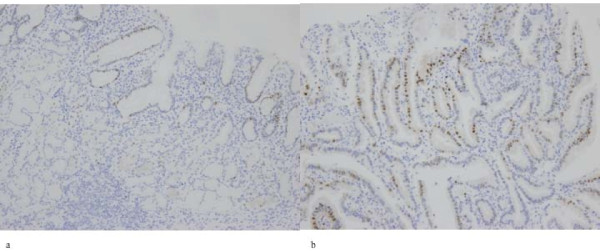
**Example of gastric intramucosal differentiated-type cancer**. Whereas the index score for cyclin D1 expression is 16 in the gastric cancer mucosa (b), the score is 4 in its surrounding non-neoplastic mucosa (a). The tumor that is shown in figure 1b is regarded as well differentiated adenocarcinoma by Japanese pathologists, although this tumor is considered gastric adenoma or dysplasia by Western pathologists.

The proportion of tumor cells per section was assessed by eye and allocated a score as summarized in Additional file 2A. A total of 500 cells were counted in random fields from representative areas of the lesion. For all markers, only nuclear staining was considered positive. The staining intensity was estimated on a 5-point scale (0 to 6+) and a score was allocated according to the intensity (Additional file 2B). The staining index was a simple multiplicative product of the cellularity and staining scores. Next, high expression for tumor cells was recorded if the staining index was higher in the tumor cells than in its surrounding non-neoplastic tissue. In contrast, low expression for tumor cells was recorded if the staining index was lower or equal in the tumor cells than in the non-neoplastic tissue. For example, a representative figure is shown in Figure 1. The original criteria were described previously [22], and the present criteria were used with minor modifications. Two sections were stained and values were based on the mean of the 2 individual results.

### Statistical analysis

Results are given as median or mean values. Statistical analysis used the Tamhane Test for comparisons among 3 groups and chi-square tests for comparisons between 2 groups. Statistical significance was established at the p < 0.05 level.

## Results

### Clinico-pathological characteristics

As shown in Additional file 3, of the 190 gastric intramucosal differentiated-type cancers, 23 (12.1%) were of the gastric phenotype, 85 (44.7%) were the intestinal phenotype and 79 (41.6%) were classified as mixed phenotypes. Only 3 (1.6%) tumors were of the unclassified type. Clinico-pathological findings based on the mucin phenotypes are also listed in Additional file 3. There were no significant differences for age, tumor size, location, microscopic type or histological type between the 3 phenotypes. However, for the gastric and mixed phenotypes, the incidences of high grade tumors were significantly higher for the gastric and mixed phenotypes than the intestinal phenotype (p < 0.01 and p < 0.05, respectively). In addition, the male/female ratio for the mixed phenotype was ower than for the other types (p < 0.05).

In the present study, gastric phenotypes were classified into 3 sub-phenotypes: MUC5AC (+)/MUC6 (+), MUC5AC (+)/MUC6 (-) and MUC5AC (-)/MUC6 (+). Fifteen (65.2%) had the MUC5AC (+)/MUC6 (+) pattern, 5 (21.7%) had the MUC5AC (+)/MUC6 (-) pattern and 3 (13.1%) were subclassified as the MUC5AC (-)/MUC6 (+) pattern. Among the 3 gastric sub-phenotypes, no clinicopathological differences were found. In contrast, intestinal phenotypes were sub-divided into small intestinal (N = 67) and colonic (N = 18) phenotypes. There were no significant differences between them for age, tumor size, location, macroscopic type or histological type.

### Expressions of cell cycle-related proteins by mucin phenotypes

No p53 overexpression was found in any surrounding non-neoplastic mucosa. Overexpression of p53 was observed in 33 of 190 cases that we examined (17.4%), and was more frequently seen in the gastric phenotype (10/23, 43.5%) compared with the other 2 types (intestinal phenotype, 13/85, 15.3% and mixed phenotype, 9/79, 11.4%). Although the difference between the gastric and intestinal phenotypes did not reach statistical significance (p = 0.054), the gastric and mixed phenotypes did show a significant difference (p < 0.05). In addition, no p53 overexpression was found in low-grade cancers (0/26). In contrast, p53 was overexpressed in 11 of 103 intermediate-grade cancers (10.7%) and 22 of 61 high-grade cancers (36.1%). Significant differences between high-grade cancer and low-grade or intermediate-grade cancers were found (p < 0.01).

p21 was primarily expressed in the superficial epithelium of the intestinal metaplastic gland and the gastric foveolar epithelium. The frequency of reduced p21 expression was 59 of 190 tumors (31.1%). Reduced p21 expression was commonly observed in the gastric, intestinal and mixed phenotypes. There was no significant difference for reduced p21 expressions between the 3 phenotypes.

Expression of p27 was confined to the superficial epithelium and the base of the intestinal metaplastic gland. The frequency of reduced p27 expression was very low in the gastric cancers that we examined (12/190, 6.3%). In contrast, the frequency for p27 overexpression was observed in 53 of 190 tumors (27.9%). The frequency of p 27 overexpression was higher in the intestinal phenotype (31/85, 36.5%) than in the gastric (6/23, 26.1%) and mixed phenotypes (16/79, 20.2%). However, these differences were not significant.

Cyclin D1 was expressed at the lower half of the intestinal metaplastic gland. In the gastric cancers examined, 27 of 190 cases (14.2%) showed overexpressed Cyclin D1. Cyclin D1 was overexpressed in the gastric phenotype for 7 of 23 cases (30.4%). By comparison, overexpression of cyclin D1 was found in 11.8% (10/85) and 12.7% (10/79) of the intestinal and mixed phenotypes, respectively. No significant difference was found between the gastric phenotype and the other types.

Expression of cyclin A was primarily confined to the lower half of the intestinal metaplastic gland. Positive staining for cyclin A was detected in 42.1% (80/190). The frequency of cyclin A overexpression was higher in the gastric phenotype (16/23, 69.6%) than in the intestinal phenotype (25/85, 29.4%; p < 0.01). However, no significant difference was found between the gastric and mixed phenotypes (39/79, 49.4%).

Non-neoplastic glands were mostly negative for cyclin E. In the gastric cancers examined, the frequency of cyclin E overexpression was very low. No significant difference between the 3 mucin phenotypes was seen.

In this study, only the nuclear expression for β-catenin was regarded as positive. No nuclear expression of β-catenin was observed in the surrounding non-neoplastic mucosa, except for pyloric glands. Most pyloric glands expressed nuclear β-catenin. Nuclear β-catenin expression was found in 80 of 190 tumors (36.8%). Although the intestinal phenotype (44/85, 51.8%) showed the highest frequency for nuclear β-catenin among the 3 phenotypes, the difference was not significant.

**Figure 2:**
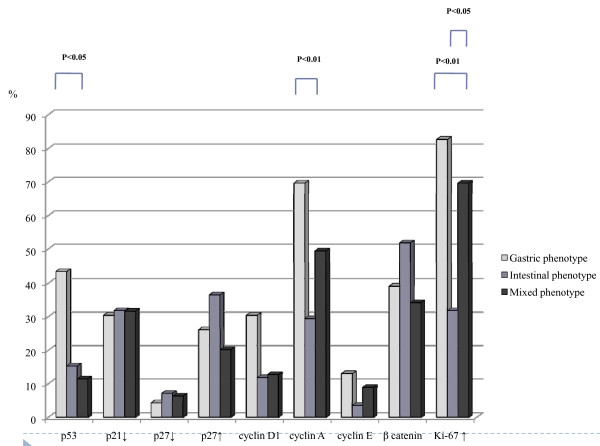
**Frequencies of cell cycle-related protein expressions in gastric, intestinal and mixed phenotypes**. Gastric phenotype cancers are characterized by p53 and cyclin A overexpressions. Although the frequency of p27 overexpression between the intestinal phenotype and other phenotypes did not reach statistical difference, this may be characteristic of the intestinal phenotype. Reduction of p21 and accumulation of β-catenin are commonly observed in the 3 phenotypes. Ki-67 positive rate is higher in the gastric phenotype or mixed phenotype than in the intestinal phenotype. Statistical comparisons used Tamhane tests for all 3 phenotypes.

**Figure 3:**
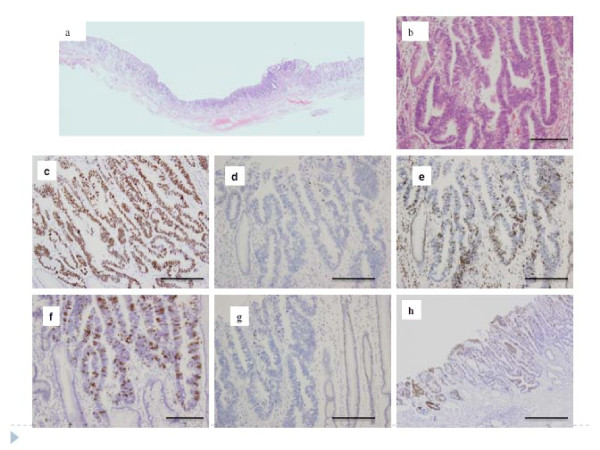
**A representative example of intestinal intramucosal differentiated-type cancer of the gastric phenotype**. a. Low power view of tumor tissue. b. High power view of tumor tissue. c. p53 overexpression was seen. d. p21 was reduced. e. p27 was overexpressed. f. Overexpression of cyclin A. g. Low expression of cyclin D1. h. Only Muc2 was expressed in this tumor, suggesting intestinal phenotype.

A high proliferation rate of tumor cells was shown in more than 30% Ki-67 PR in most of the gastric cancers that we examined. According to our criteria, high proliferation was found in 101 of 190 tumors (53.2%). High proliferation of tumor cells was more frequently observed in the gastric and mixed phenotypes compared with the intestinal phenotype (p < 0.01 and p < 0.05). We have summarized these results for cell cycle-related proteins based on mucin phenotype in Figure 2. In addition, a representative example is shown in Figure 3.

## Discussion

In spite of a decreasing trend in incidence, gastric cancer remains an important medical problem throughout the world, and it is the most common neoplasm in Japan. Intramucosal cancer is defined as a tumor at the most early stage of gastric cancer. It is essential to examine intramucosal cancer for evaluating the pathogenesis of gastric cancer. It is widely accepted that a cancerous cell arises due to abnormalities of cell cycle regulation, which is comprised of several cell cycle-related proteins, such as cyclins, cyclin dependent kinase inhibitor, p53 and β-catenin. In addition, recent studies have shown that distinct clinico-pathological findings for gastric cancer are characterized by mucin expressions. We studied abnormal expressions of cell cycle-related proteins that promote gastric carcinogenesis based on mucin phenotypes in gastric intramucosal differentiated-type cancers.

In the present study, immunohistochemistry was used to investigate the expression levels of cell cycle-related proteins. Immunopositivity for cell cycle-related proteins of tumor tissue was determined by comparing the levels between tumor tissue and its surrounding non-neoplastic tissue. According to these criteria, the level of immunopositivity of tumor tissue may differ for each case. However, separating the parameters within these cancers into "low" and "high" categories based on comparisons of these factors with non-neoplastic tissue may be an unconventional method. The "non-neoplastic" tissue includes gastritis (chronic or chronic active) and/or intestinal metaplasia; the parameters in these various inflammatory or metaplastic tissues could vary considerably. It may be more meaningful to use an absolute rate (i.e., the actual %), rather than this internal relative value. However, we consider that this immunohistochemical criterion is important for finding the potential activities of cell cycle-related proteins at an early stage of gastric differentiated-type cancers, given that the non-neoplastic tissue surrounding tumor tissue is closely associated with tumor development.

It has been believed among pathologists that the gastric phenotype of gastric cancer shows low-grade atypia in gastric intramucosal differentiated-type cancers (19). However, in the present study, in the gastric intramucosal differentiated-type cancers that we examined, the gastric phenotype showed high-grade atypia. Although the reason remains unknown, one possible reason is that a gastric phenotype showing low-grade atypia, which resembles gastric foveolar epithelium histologically, may have been included in previous studies. In the present study, only one such tumor was observed. In addition, these differences could be due to the number of samples or to ethnic or etiologic factors. It is well known that p53 overexpression is correlated with tumor grade. In the present study, we suggest that it is reasonable to show a high frequency of p53 overexpression for the gastric phenotype when compared with other phenotypes.

In this study, p53 overexpression was more frequently found in the gastric phenotype than in the intestinal or mixed phenotypes. This finding suggests that p53 overexpression plays an essential role, at least in the early development of gastric phenotype cancer, although previous studies have shown progressive increases in p53 overexpression in gastric cancers from the early to advanced stages [12].

In this study, reduced p21 expression was observed in approximately 30% of the 3 mucin phenotypes. Previous studies have shown that low expression of p21 is correlated with patient prognosis in gastric cancer [5,6]. However, little is known regarding p21 expression by gastric cancers based on mucin phenotypes. In addition, any association of p21 expression with tumor stage (tumor depth) remains unknown. Our finding suggests that reduced p21 expression plays a common role in the early development of the 3 mucin phenotypes of gastric differentiated-type cancers. However, a previous study suggested that a progressive decrease of p21 expression occurred in an advanced phase of gastric differentiated-type cancer from the early phase [6]. p21 expression also correlated with the expressions of other cell cycle-related proteins, such as p27 and Ki-67, possibly through a p53-dependent pathway [1,5,6]. It has been hypothesized that p21 co-operates with p53 and, as a result, the cell cycle is controlled through a p53-dependent pathway [1,5]. Furthermore, it is believed that p21 is also inhibited by co-operation with p27 [1,5]. In the present study, however, we found neither a correlation of p21 reduction with p27 reduction nor an inverse correlation of p27 reduction with p53 overexpression (data not shown). We suggest that a different pathway exists for controlling the cell cycle in human gastric cancers.

It is well known that p27 is a prototypical tumor suppressor gene [1-3]. However, a recent study has shown that, whereas in some cases, p27 inhibits the cell cycle, in other cases it promotes progression to the next phase of the cell cycle [23]. It has been reported that either a loss or a low expression of p27 is correlated with tumor progression or patient prognosis [24,25]. In contrast to other studies, we showed that low expression (reduction) of p27 had a very low frequency in our series of gastric cancers. According to the present findings, reduced p27 expression plays a minor role in the development of differentiated type gastric cancers.

In contrast, overexpression of p27 was frequently found in the intestinal phenotype compared with the other 2 phenotypes, although the difference between them did not reach a statistically significant level. Whether or not this overexpression of p27 is associated with cell cycle inhibition or cell cycle progression is not known, although several explanations are suggested. First is that p27 that is overexpressed for suppression of cell cycle progression in tumor cells results from a negative feedback mechanism. Conversely, a second explanation is that overexpression of p27 is positively associated with the progression of the cell cycle for tumor cells; that is, p27 acts as an oncogenic factor [23]. In addition, a recent study has shown that expression of p27 is regulated by expression of Skp2, which is a ubiquitin ligase subunit of p27 [26]. According to this report, expression of p27 was inversely correlated with Skp2 expression.

Cyclin D1 overexpression only showed a low frequency in the early stage of gastric differentiated type cancers (27/190, 14.2%). However, although the differences did not reach a significant level, the frequency of cyclin D1 overexpression was higher in the gastric phenotype than in the intestinal and mixed phenotypes. Cyclin D1 was previously studied in several series of gastric cancers, with a reported frequency of 20~56% of overexpression being reported [27-29], different from our results. As a possible reason for these differences of cyclin D1 overexpression, although early differentiated-type cancers, except for gastric phenotype, do not require an increase of cyclin D1, once the tumor has developed, the tumor cells need a high level of cyclin D1 for tumor progression. This suggests that cyclin D1 overexpression is correlated with patient prognosis in gastric cancers [28].

Overexpression of cyclin A was also more prevalent in the gastric and mixed phenotypes compared with the intestinal phenotype. This suggests that overexpression of cyclin A plays a major role in tumorigenesis of gastric and mixed phenotypes. Thus, in gastric cancers, acquisition of gastric mucin expression may be associated with cyclin A overexpression, which then results in high tumor proliferation. In addition, cyclin A was closely associated with patient prognosis or tumor aggressiveness [8,9]. This may indicate that cyclin A overexpression is related to both tumor development and progression in approximately half of differentiated-type gastric cancers.

In the present study, cyclin E overexpression showed low frequencies in the 3 phenotypes. In contrast, previous studies have shown that cyclin E overexpression was correlated with poor outcome [4,10], although contrasting results have been suggested [30]. The present results indicate that cyclin E overexpression plays no essential role in the early tumorigenesis of differentiated-type gastric cancers. In addition, overexpression of cyclin E may primarily promote the progression of gastric cancers. These findings suggest that the high proliferation of the gastric phenotype may be characterized by the overexpression of cyclin D1 and/or cyclin A, and that the mixed phenotype is associated with cyclin A overexpression.

It is well known that β-catenin expression upregulates the cell cycle and plays a central role in the Wnt signal transduction pathway [14,15]. In the present study, it is interesting to note that β-catenin expression was commonly observed in the early differentiated-type gastric cancers that we examined. It is already accepted that β-catenin expression is critical for colonic adenoma development [31,32]. The present results and previous studies regarding colorectal adenoma indicate that β-catenin expression may be a common alteration in the early development of gastrointestinal tumors [31]. This could be explained by the findings that, in gastric cancers, gene promoters associated with the Wnt signal pathway are highly methylated [32-34], which results in the nuclear accumulation of β-catenin expression.

## Conclusions

**Figure 4:**
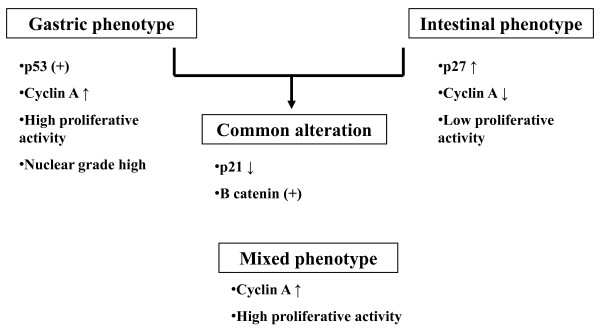
**A new hypothesis for tumorigenesis of gastric intramucosal differentiated-type cancer as defined by the mucin phenotype**.

In summary, we propose that the cellular mucin phenotypes of intramucosal differentiated-type adenocarcinomas of the stomach are dependent on distinct cell cycle-related alterations, and that the clinico-pathological findings result from different pathways based on mucin expression: gastric, intestinal and mixed phenotypes. Figure 4 illustrates a novel carcinogenesis model for intramucosal differentiated-type adenocarcinomas that relies on their mucin phenotypes. Based on abnormalities of cell cycle-related proteins, overexpressions of p53 and cyclin A characterize gastric phenotype cancers, whereas overexpression of p27 may be associated with the development of intestinal phenotype cancers. In contrast, mixed-phenotype cancers are characterized by cyclin A overexpression. In addition, mucin phenotypes appear to be characterized by nuclear grade and cell proliferative activity. Three of 190 tumors were of the unclassified type, and this tumor molecular characteristic is not identified. Finally, little is known regarding the distinct molecular alterations that are involved in the pathogenesis of gastric cancer. Further studies will be necessary to address this question.

## Competing interests

The authors declare that they have no competing interests.

## Authors' contributions

MT, ME and HM carried out the clinicopathological studies, YS and YM carried out the immunoassays, NT carried out statistical advice, MT carried out molecular genetic advice, KS participated in the sequence alignment and drafted the manuscript. All authors read and approved the final manuscript.

## Pre-publication history

The pre-publication history for this paper can be accessed here:

http://www.biomedcentral.com/1471-230X/10/55/prepub

## Supplementary Material

Additional file 1 **Table S1 contained clinicopathological findings of intramucosal cancers**. Click here for file

Additional file 2 **Table S2 contained criteria for determination of score of tumor cells**. Click here for file

Additional file 3 **Table S3 contained clinicopathological findings of gastric intramucosal differentiated-type cancer based on mucin phenotype**. Click here for file
