# Supplementary material for: Analysis of cell cycle-related proteins in gastric intramucosal differentiated-type cancers based on mucin phenotypes: a novel hypothesis of early gastric carcinogenesis based on mucin phenotype
Source: BMC Gastroenterol. 2010 Jun 7;10:55. doi: 10.1186/1471-230X-10-55 (PMC2903504; doi:10.1186/1471-230X-10-55)
Supplement: Additional file 1 — Table S1 contained clinicopathological findings of intramucosal cancers. [file 1471-230X-10-55-S1.DOC]

Table 1：Clinicopathological findings of intramucosal cancers

Intramucosal differentiated-type cancer(％)

　　　　Total 　　　　　　　　　　　　　　　　　　　　　　　　　　190

　　　　Sex (Man/Woman) 　　　　　　　　　　　　　　　　　　　　 129/61

　　　　Age (mean)　　　　　　　　　　　　　　　　　　　　　　　　49-86 (70.8)

　　　　Size (mm，mean)　　　　　　　　　　　　　　　　　　　　　 2-80 (14.0)

　　　　Location (P/D)　　　　　　　　　　　　　　　　　　　　　　 17/173

　　　　Macroscopic type

　　　　Elevated type 　　　　　　　　　　　　　　　　　　　　　 108 (55.8)

　　　　flat type 5 (2.8)

　　　　Mixed type 10 (5.5)

　　　　Depressed type 67 (35.9)

　　　　Histological type

　　　　WDA 170 (89.5)

　　　　MDA 18 (9.5)

　　　　PA 2 (1.0)

WDA, well differentiated adenocarcinoma；MDA，moderately differentiated adenocarcinoma；PA，papillary adenocarcinoma
