# Supplementary material for: Analysis of cell cycle-related proteins in gastric intramucosal differentiated-type cancers based on mucin phenotypes: a novel hypothesis of early gastric carcinogenesis based on mucin phenotype
Source: BMC Gastroenterol. 2010 Jun 7;10:55. doi: 10.1186/1471-230X-10-55 (PMC2903504; doi:10.1186/1471-230X-10-55)
Supplement: Additional file 2 — Table S2 contained criteria for determination of score of tumor cells. [file 1471-230X-10-55-S2.DOC]

Table2：Criteria for determination of score of tumor cells

A B

　　　80％≦PC　　　　　　　　　　　　6 Strong 6

　　　50％≦PC＜80％　　　　　　　　　5 Intermediate 4

　　　30％≦PC＜50％　　　　　　　　　4 Weak 2

　　　10％≦PC＜30％　　　　　　　　　3 Very weak 1

　　　PC＜10　　　　　　　　　　　　　2 Negative 0

　　　Scattered　　　　　　　　　　　 1

　　　Negative　　　　　　　　　　　　 0
