# Supplementary material for: Analysis of cell cycle-related proteins in gastric intramucosal differentiated-type cancers based on mucin phenotypes: a novel hypothesis of early gastric carcinogenesis based on mucin phenotype
Source: BMC Gastroenterol. 2010 Jun 7;10:55. doi: 10.1186/1471-230X-10-55 (PMC2903504; doi:10.1186/1471-230X-10-55)
Supplement: Additional file 3 — Table S3 contained clinicopathological findings of gastric intramucosal differentiated-type cancer based on mucin phenotype. [file 1471-230X-10-55-S3.DOC]

Table3：Clinicopathological findings of gastric intramucosal differentiated-type cancer based on mucin phenotype

G phenotype(％) I phenotype(％) M phenotype(％) U phenotype(％)

Total 23 (12.1) 85 (44.7) 7 9(41.6) 3 (1.6)

Sex (Man/Woman) 15/8 66/19 47/32 ＊ 0/2

Age (mean) 51-82 (70.7) 55-86 (71.2) 49-85 (70.9) 66-72 (69.7)

Size (mm，mean) 3-24 (15.1) 2-52 (13.5) 2-80 (14.3) 6-37 (20.3)

Location (P/D) 3/20 10/75 4/75 1/2

Macroscopic type

Elevated type 11 (47.8) 57 (67.1) 37 (46.8) 3 (100)

Flat type 1 (4.3) 2 (2.3) 2 (2.5) 0

Mixed type 3 (13.0) 3 (3.5) 4 (5.1) 0

Depressed type 8 (34.8) 23 (27.1) 36 (45.6) 0

Histological type

WDA 18 (78.3) 84 (98.8) 66 (83.5) 2 (75)

MDA 3 (13.0) 1 (1.2) 13 (16.5) 1 (25)

PA 2 (8.7) 0 0 0

Nuclear grade

Low 1 (4.4) 18 (21.2) 7 (8.9) 0

Intermediate 7 (30.4) 56 (65.9) 40 (50.6) 0

High 15 (65.2)＊＊ 11 (12.9) 32 (40.5)＊ 3 (100)

G type，Gastric type；I type,Intestinal type；M type，Mixed type；U type，Unclassified type

＊ p＜0.05 ＊＊ P＜0.01
